# The complete mitochondrial genome of the *Odorrana schmackeri* (Anura, Ranidae)

**DOI:** 10.1080/23802359.2016.1144112

**Published:** 2016-03-28

**Authors:** Xingjiang Bu, Lijuan Zhang, Kexin He, Yanmei Jiang, Liuwang Nie

**Affiliations:** Anhui Provincial Key Laboratory of the Conservation and Exploitation of Biological Resources, College of Life Sciences, Anhui Normal University, Wuhu, PR China

**Keywords:** Mitogenome, *Odorrana schmackeri*, Ranidae

## Abstract

The complete mitochondrial genome of *O. schmackeri* has been sequenced and characterized in this study. The mitogenome is a circular molecule of 18*^ ^*610 bp in length, containing 13 protein-coding genes (PCGs), two ribosomal RNA (rRNA) genes, 21 transfer RNA (tRNA) genes and a non-coding D-loop region (control region). Its gene arrangements are identical to the typical neobatrachian-type except for the loss of *tRNA^His^* gene. Our data provide a useful resource for the phylogenetic studies of genus *Odorrana*.

The Chinese piebald odorous frog (*Odorrana schmackeri*) is widely distributed in southern and south-central China at 200–1400 m elevation (Frost 2015). The specimen of *O. schmackeri* was captured from Huangshan, Anhui province in China (30°06′N, 118°09′E; 595 m elevation) and stored in Anhui Provincial Key Laboratory of the Conservation and Exploitation of Biological Resources from Anhui Normal University. Total genomic DNA was extracted from *O. schmackeri* muscle tissue using the standard phenol–chloroform protocol, as described by Sambrook and Russell (2001), and the complete mtDNA of *O. schmackeri* was amplified and sequenced using 17 primer pairs.

In this study, we determined the complete mitochondrial genome sequence of *O. schmackeri*, which is 18*^ ^*610 bp in size (GenBank accession no. KP732086). The circular mitogenome contains 36 genes, including 13 typical protein-coding genes, 21 tRNA genes (*tRNA^His^*gene lacked), two rRNA (*12S rRNA* and *16S rRNA*) genes and a control region. Most genes were encoded on the heavy strand (H-strand) except for *ND6* gene and eight tRNA genes (*tRNA^Pro^*, *tRNA^Gln^*, *tRNA^Ala^*, *tRNA^Asn^*, *tRNA^Cys^*, *tRNA^Tyr^*, *tRNA^Ser(UCN)^* and *tRNA^Glu^*) which were encoded on the L-strand.

The non-coding regions in the *O. schmackeri* mtDNA included the control region and some intergenic spacers. The control region was located between *Cyt b* and *tRNA^Leu (CUN)^* genes, with a 2769 bp length. Two long (167 bp and 229 bp) and several short (1–67 bp) non-coding sequences are dispersed in the *O. schmackeri* mtDNA. The putative origin of light-strand replication (OL) (30 bp) is situated between the *tRNA^Ala^* and *tRNA^Asn^* genes instead of between *tRNA^Asn^* and *tRNA^Cy^* as in most vertebrates in the WANCY tRNA cluster (Su et al. 2007; Jiang et al. 2015; Sun et al. 2015; Yan et al. 2016).

The 13 identified PCGs were 11*^ ^*299 bp in total length (168–1794 bp). Nine of the 13 protein-coding genes initiated with ATG as the start codon, while *COI* and *ATP6* began with ATA, and *ND2* and *ND4L* started with ATT. Stop codons were variable for all protein-coding genes. Six genes (*ATP8*, *ND4L*, *ND4*, *Cyt b, ND2* and *ND5*) used the common TAA and TAG as the stop codon, whereas, *COI* and *COII* ended with AGA, and *ND6* stopped with AGG. Incomplete stop codons (T– –) were found in *ND1*, *ATP6*, *ND3* and *COIII*.

The 21 tRNA genes ranged in size from 64 bp (tRNA^Cys^) to 73 bp (tRNA^Asn^ and *tRNA^Leu (UUR)^*). In the new mitogenome, the notable feature is the loss of *tRNA^His^*, we were unable to find the potential *tRNA^His^* gene at any other location in the mitogenome of *O. schmackeri*. In contrast to the PCGs, loss of tRNA genes is relatively more frequent during the evolution of animal mtDNAs (Zhang et al. 2009). Nevertheless, the loss of *tRNA^His^* gene was discovered for the first time in anurans. The predominant explanation for the mitochondrial gene loss is the gene replacement hypothesis (Adams & Palmer 2003).

The complete mitogenomes sequences of *O. schmackeri* and other individuals belonging to Ranidae were used for phylogenetic analysis, with setting *Microhyla ornata* as outgroup (Figure 1). Maximum-likelihood method (ML) was used to examine the phylogenetic position of *O. schmackeri* applying RAxML (7.2.6) (Stamatakis 2007). It appeared that *O. schmackeri*, *O. tormotus*, *O. margaretae* and *O. ishikawae* formed a monophyletic group. These data provide a powerful tool for systematic analysis of genus *Odorrana* and family Ranidae.

**Figure 1.**
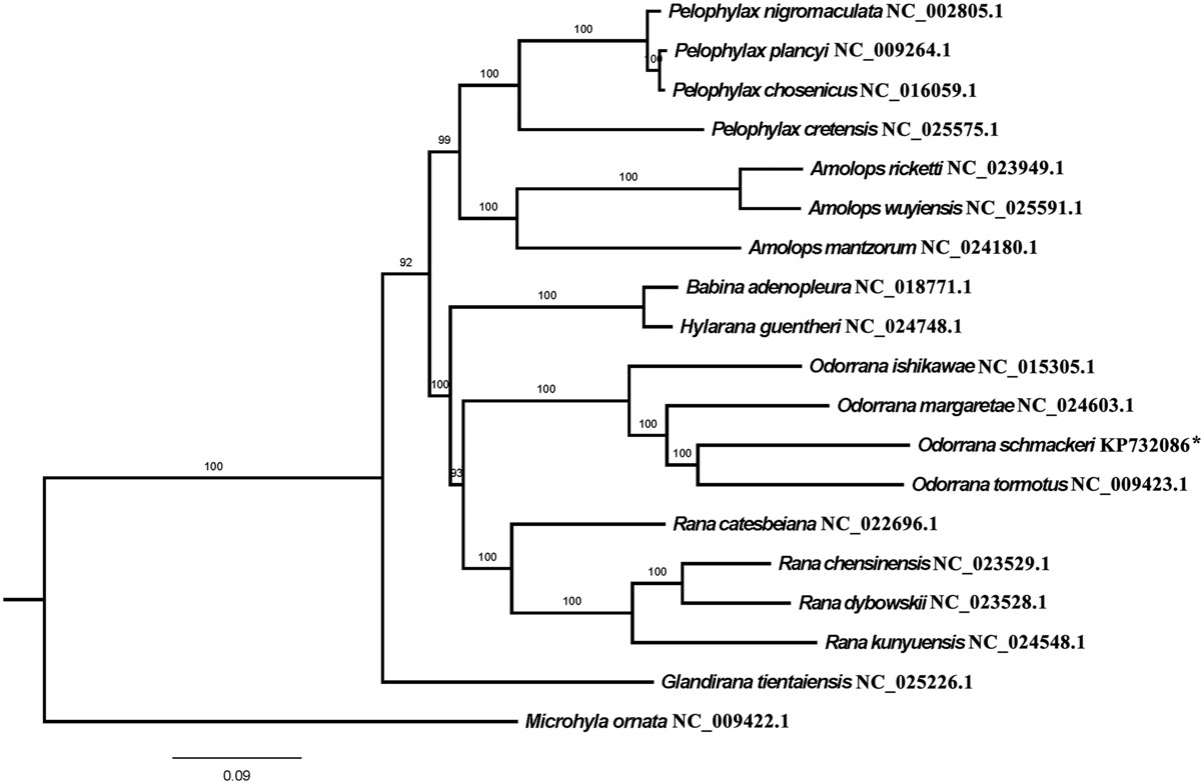
ML phylogeny of Ranidae species based on the complete mitochondrial genomes. The asterisk indicates the sequence generated in this study.
